# Event and Comment

**Published:** 1907-02-15

**Authors:** 


THE
### DENTAL REGISTER.

Vol. LXI. February 15, 1907. No. 2.

### EVENT AND COMMENT.

### Evils of the Tooth Brush and Powder.

Prof. W. D. Miller has been making an extensive research on the causes of the wasting away of the tooth structure, such as is frequently observed on the labial and buccal surfaces, taking the form of clear cut V shaped notches or cavities, and sometimes involving the enamel of the entire labial surface of one or more teeth. We have long been in doubt as to whether this was a purely mechanical result or was due to a chemical erosion, or to a combination of the two. Prof. Miller publishes in the January 1907, *Cosmos* the result of his research on the mechanical theory, and he sums up his finding in this statement at the end of this article: "I have been more and more confirmed in the opinion that the majority of cases of wasting met with in daily practice are due chiefly to the action of the brush in conjunction with tooth powder, and that compared with this one, other mechanical agents are of minor importance." He arrives at this conclusion after a long research in which he made close investigation of many cases of marked loss of the hard tooth tissues not attributable to Caries, in all of which he was able to conclusively decide that the wasting was due to an excessive use of the brush with powders which were overloaded with grit. He further confirms the research by one made on teeth out of the mouth, using the brush and various grits of powder and the usual method of brushings.

The results shown certainly justify the conclusions he reaches and it looks as though we shall have to modify our mouth toilet as well as instructions to patients. When we think of it comparatively, we shall have to conclude that few people brush their teeth enough to produce serious abrasion of the enamel. We do not often see cases of abrasion on all or even a large number of the teeth. It is not uncommon to see the cuspid or other prominent teeth slightly abraded especially on the side where the patient first applies the brush filled with powder, or where he naturally applies it most vigorously and for the longest time.

We recently saw in the mouth of a gentleman who makes a tooth powder, in which there is no grit at all, it being a vegetable powder, the typical V shaped abrasion of all the lower front teeth, which we have no doubt was caused wholly by brushing excessively with this gritless powder. We regret now that we did not inspect the teeth more carefully; but the gentleman assured us that these teeth at one time had a considerable coating of caludus which he had removed by brushing across the teeth with a flat and stiff tooth brush with his powder. Dr. G. V. Black recently asked us how many cases of erosion we could recall in our practice, and we were compelled to say that we could not remember more than a half dozen distinct and extensive cases. He said our statement tallied with the answer he had received from the large majority of practitioners to whom he had put the same question. And yet he added that one practitioner had agreed to present him plaster models of over fifty cases from his practice, and that he was likely to do so, at the rate he was sending them in. If Prof. Miller's conclusions are carefully considered by the gentleman we mention, and by Dr. Black's friend, it may be that they will think it is time to modify their tooth powder formula or their instructions on brushing the teeth.

Notwithstanding Prof. Millers conclusions and the cases we have cited, we believe it will be found after all that this wasting of the tooth tissues is due to the manner of using the brush and powder, rather than to the nature of these necessary toilet articles. An excessively gritty powder on a very stiff or thickly set brush, and used with all one's might across the surfaces of the teeth twice a day, undoubtedly should wear even so hard a substance as tooth enamel. But a well selected brush, adapted to each individual case, and a finely ground powder thoroughly incorporated in the wet bristles, can, by the proper rotary movements, be forced into the tooth embrasures and sulci so as to clean away all mucoid deposits without the slightest danger of abrading the enamel.

**Prof. Black Banqueted.**

The St. Louis Society of Dental Science tendered Dr. G. V. Black a banquet January 15th, 1907, and judging from the menu and list of toasts and eminent speakers it surely must have been a great privilege to have been present. There can be no question about the merits of such a testimonial in this instance, as the object could not have been more worthy. It was also particularly appropriate for this society to tender the testimonial. The society is a new one, the result of a consolidation of the two societies which have existed for several years in St. Louis, and therefore representative of the entire profession of St. Louis and that section in which Dr. Black began his professional career. It was to the old dentists of St. Louis in particular that Dr. Black owes the incentive to take up the scientifie work in behalf of his profession that has made his name honored throughout the entire dental world. When Dr. Black was a young man there was in St. Louis a coterie of broad-minded practitioners and men with right professional instincts who were not only expert clinicians, but they did much of the pioneer scientific work of that time, and more to stimulate others to take it up. Dr. Black grew up in this atmosphere, and he not only inherited what naturally came to him as a result of association with the men of his time, but he began to think and work on his own account, and he has improved his opportunity since then endeavoring to make plain to the profession more of the mysteries of our calling than any other man in this country. He has merited not only the honors of his neighbors, but of the entire professional world. It is perhaps safe to say that few other men have gained the respect and love of the entire profession to a greater extent, and the work that he has done will live long after him.

### Commission Fees.

The American Society of Orthodontists in convention assembled in New York City, December 29th, 1906, passsed the resolutions which we print below, which if accepted by those practicing this specialty, will be a step forward in professionalism to be generally endorsed and commended.

RESOLVED: That in the opinion of the members of the American Society of Orthodontists, the practice of paying a commission, honorarium, or any sort of a fee, as a consideration for the reference of a patient is both unwarrantable and unprofessional; and be it

Resolved, That the payment of any such commission, honorarium, or fee, by any member of this Society, shall be sufficient cause for the expulsion of said member, by vote of the Society after conviction; and further be it

Resolved, That in case of co-operation in the care of a patient between a general practitioner and an Orthodontist, there shall be no division of fees, but each man shall render a separate bill for his personal services.

Viewed from the moral standpoint, there can be no question but this action is a righteous one, therefore it should be accepted by every true professional man. The practice of Orthodontia, as a specialty, has flourished because of the fact that the general practitioner has helped it on by referring cases for the liberal commission which he received for a service that he had no part in. It was easy money and no responsibility. It remains to be seen whether the great advances this specialty has made will continue under this handicap. It is to be hoped that it has sufficient momentum to keep its pace, and that the profession will even more cordially support it than before.

